# The effect of an environment-based educational program on adolescents’ pro-environmental behaviors and ecological footprint awareness: a quasi-experimental study

**DOI:** 10.1590/1806-9282.20260625

**Published:** 2026-09-21

**Authors:** Neslihan Başaran, Busra Altinel

**Affiliations:** 1 Selçuk University, Institute of Health Sciences, Public Health Nursing – Konya, Turkey.; 2 Selçuk University, Faculty of Nursing, Public Health Nursing – Konya, Turkey.

**Keywords:** Adolescent, Health education, Environment, Behavior, Health knowledge, attitudes, practice

## Abstract

**OBJECTIVE:**

The aim of this study was to examine the effect of an Environment-Based Education Program applied to adolescents on their pro-environmental behavior and ecological footprint awareness.

**METHODS:**

This research, conducted in a quasi-experimental design with a pre-test-post-test control group in non-randomized samples, was carried out with 10th-grade students attending a vocational high school in the Central Anatolia region of Turkey. The study included 52 students in the intervention group and 52 students in the control group. The Environment-Based Education Program, consisting of three weeks and three sessions, was applied to the students in the intervention group. Data were collected using a Personal Information Form, the Pro-Environmental Behavior Scale, and the Ecological Footprint Awareness Scale. Chi-square, dependent samples t-test, and independent samples t-test were used in the analysis of the data.

**RESULTS:**

After the applied Environment-Based Education Program, it was found that the mean total scores on the Pro-Environmental Behavior Scale of the intervention group increased in the post-test compared to the pre-test, and the difference was statistically significant (p<0.001). No significant difference was found between the intervention and control groups in ecological footprint awareness scores (p>0.05).

**CONCLUSION:**

The Environment-Based Education Program applied to adolescents was found to increase their pro-environmental behaviors. In light of these results, environmental education programs should be organized periodically for adolescents, and school visits should be planned by public health nurses.

## INTRODUCTION

Climate change-related environmental deterioration is expected to cause approximately 250,000 additional deaths per year between 2030 and 2050^1^. Rapid socio-economic development contributes to biodiversity loss and environmental degradation^2^, underscoring the continued importance of environmental education for sustainability and biodiversity conservation^3^. Environmental education aims to enhance awareness and understanding of the natural environment while fostering the attitudes, skills, and motivation needed to address challenges such as climate change, pollution, and environmental degradation^4^.

Environmental education programs targeting adolescents are particularly important, as they can strengthen beliefs in personal environmental impact and promote behavior change^5^. Such programs have been associated with more positive environmental attitudes and behavioral intentions, as well as improvements in self-confidence, social interaction, and academic motivation^6^. In this context, developing pro-environmental behaviors - defined as actions aimed at protecting or minimizing harm to the environment - is essential^7^. Previous studies have shown that environmental education positively influences these behaviors, often mediated by increased environmental responsibility^8,9^.

Promoting sustainable living also requires raising awareness of ecological footprints, encouraging individuals to avoid overconsumption and adopt environmentally responsible practices^10^. Increasing ecological footprint awareness is considered an effective way to foster sustainability and environmental responsibility within society^11^. Accordingly, several studies have reported that environmental education activities positively influence ecological footprint awareness and environmental consciousness^10,12,13^. It is recommended that such programs be tailored to adolescents’ developmental characteristics to maximize effectiveness^14^.

This study aimed to examine the effect of an environment-based education program on adolescents’ pro-environmental behaviors and ecological footprint awareness. The study tested the following hypotheses:

H0_1_: There is no difference between the intervention and control groups in terms of pro-environmental behavior levels.
H0_2_: There is no difference between the intervention and control groups in terms of ecological footprint awareness levels.

## METHODS

This study was registered on 28 September 2021 on ClinicalTrials.gov with the Identifier, NCT05073198.

### Study design

This study was conducted using a quasi-experimental design with a non-randomized pre-test–post-test control group.

### Study population and sample size

The study population consisted of 125 tenth-grade students attending a vocational high school in the Central Anatolia region of Turkey. Sample size was calculated using G*Power version 3.1.9.4 at a 95% confidence level, with a significance level of α=0.05 and a statistical power of 0.80. As no previous experimental study evaluating the effect of environmental education on ecological footprint awareness among adolescents using the same outcome measure was identified, the effect size (d=0.58) reported by Günşen was used as the best available estimate for sample size calculation. Although that study was conducted with pre-service preschool teachers, it was selected because the educational intervention addressed similar environmental education content and ecological footprint awareness outcomes. Based on these assumptions, the minimum required sample size was calculated as 94 participants, with at least 47 students in each group. To account for potential attrition and group separation, the sample size was increased by 10%, and the study was conducted with 104 students. To prevent contamination between students, intervention and control groups were assigned at the classroom level rather than through individual randomization. Eligible classes were allocated to the intervention or control group by lottery. Although this approach reduced the likelihood of information exchange during the intervention period, communication between students outside the classroom setting could not be completely controlled.

### Inclusion and exclusion criteria

Inclusion criteria for the study: Being in the 10th grade, being able to speak and understand Turkish. Exclusion criteria: Having prior environmental education (based on self-report in the Personal Information Form, as previous environmental education could influence baseline knowledge and awareness levels and affect the evaluation of the intervention), having any communication or medically diagnosed mental illness. Withdrawal criteria: Wishing to leave the study at any stage, not attending any of the trainings.

### Data collection

**Personal Information Form:** Prepared by the researcher in line with the literature^10,13^, the form consists of nine questions aimed at determining the basic characteristics of the students.

**Pro-Environmental Behavior Scale:** Developed to determine participants’ pro-environmental behaviors, this scale consists of 26 items on a five-point Likert scale. The scale is rated as follows: "26–46 points very low," "47–67 points low," "68–88 points medium," "89–109 points high," "110–130 points very high." The scale consists of three sub-dimensions: environmental education, economic behaviors, and recycling. The Cronbach-alpha internal consistency coefficient of the Environmental Friendly Behavior Scale was calculated as 0.832 for the scale as a whole, 0.728 for the environmental education dimension, 0.581 for the economic behaviors dimension, and 0.790 for the recycling dimension^15^.

**Ecological Footprint Awareness Scale:** This scale aims to determine the level of ecological footprint awareness. The scale consists of 46 questions in five sub-dimensions: food, transportation and housing, energy, waste, and water consumption. The Cronbach-alpha internal consistency coefficient of the Ecological Footprint Awareness Scale was determined as 0.942 for the scale as a whole, while it was calculated as 0.673 for the food dimension, 0.720 for the transportation and housing dimension, 0.876 for the energy dimension, 0.866 for the waste dimension, and 0.810 for the water consumption dimension. As the score obtained from the five-point Likert scale increases, the level of ecological footprint awareness also increases^16^.

**Figure 1:**
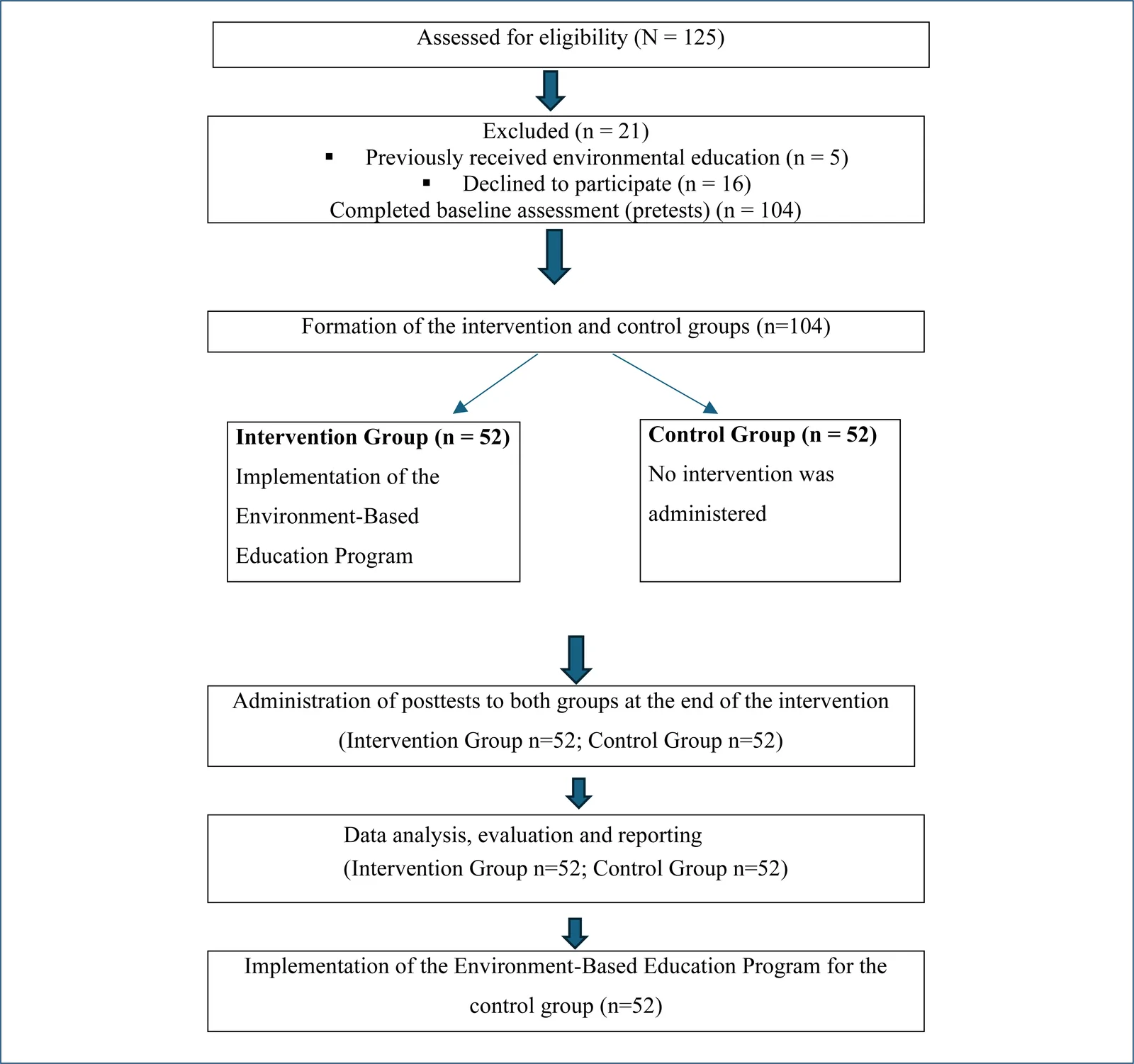
Flow diagram of the study.

**Data collection:** Data were collected by the researcher through face-to-face surveys in a classroom setting between April and May 2024 (Figure 1).

### Procedure

Before implementation, expert opinions on the Environment-Based Education Program were obtained from three faculty members holding the title of Associate Professor in the field of Public Health Nursing. The educational content was reviewed by the experts for content validity, age appropriateness, and suitability for adolescent participants. The three-session program was developed based on the scope of the educational content and the feasibility of implementation within the school schedule. The educational content was prepared following a review of the relevant literature on environmental education and ecological footprint awareness. The program sessions were delivered by the researcher and conducted according to a standardized educational plan to ensure consistency in content and implementation across all sessions. Consistency was promoted through the use of standardized educational materials and delivery procedures across all sessions.

### Intervention group

Week 1 "Environment and Environmental Problems": The first week of the Environment-Based Education Program aimed to equip participants with the skills to assess the causes and potential consequences of environmental problems, and to help them develop solutions to combat these problems. The environment and environmental problems were explained, a video prepared by a researcher who visited waste centers was shown, and suggestions were received on preventing environmental pollution.

Week 2 "Developing Pro-Environmental Behaviors": This week was planned to evaluate what pro-environmental behaviors are and what can be done to develop them. The goal was for participants to understand pro-environmental behaviors and discover how they can more effectively apply them in their own lives. The topic of pro-environmental behaviors was explained, a short documentary/animation about pro-environmental behaviors was shown, and students were asked to give an example of a pro-environmental/non-pro-environmental behavior to reinforce pro-environmental behaviors.

Week 3 "Ecological Footprint Awareness": This week aimed to evaluate the meaning of the term ecological footprint and what can be done to increase awareness on this topic. The goal was for participants to understand their own ecological footprints and discover ways to reduce their environmental impact. The training covered the topic of ecological footprint awareness, introduced projects carried out within the province, and included a Taboo game incorporating concepts discussed throughout the Environmental-Based Education Program.

Control group: No intervention was made in the control group, and students continued their normal educational processes. Final tests were collected at the end of the third week.

### Data analysis

Data were analyzed using the Statistical Package for the Social Sciences (SPSS) 29.0 software package. Data were summarized as numbers, percentages, means, and standard deviations. The normality of continuous variables was assessed using the Shapiro-Wilk test prior to parametric analyses. Chi-square and Fisher's exact tests were used to analyze the similarity between intervention and control groups. Independent samples t-tests were used to compare pre-test and post-test measurements between intervention and control groups according to dependent variables; dependent samples t-tests were used to compare pre-test and post-test measurements within intervention and control groups according to dependent variables. All statistical tests were conducted as two-tailed tests, and p<0.05 was considered statistically significant.

Ethical Considerations: Before commencing the study, ethical approval was obtained from the Selcuk University Nursing Faculty Non-Interventional Clinical Research Ethics Committee on February 23, 2024, with approval number 2024/16. Institutional permission was obtained from the Konya Provincial Directorate of National Education Provincial Ministry of National Education and the institution where the study would be conducted. In the research, the condition of "informed consent" was implemented as an ethical principle, as it is necessary to protect the rights of the individuals participating in the research. In addition, parental consent was obtained from the families of the individuals participating in the research. Furthermore, environmental education was provided to the individuals in the control group, who did not undergo any intervention, in accordance with the principle of "equality."

## RESULTS

Students in the intervention and control groups were similar in terms of sociodemographic and environmental awareness characteristics (p>0.05).

The intervention group's mean scores on the Environmental Behavior Scale, specifically the sub-dimensions of environmental education and recycling, increased in the post-test compared to the pre-test. The intervention group had significantly higher environmental education subdimension scores than the control group (p<0.001). Similarly, the intervention group's mean scores on the economic behaviors sub-dimension increased in the post-test compared to the pre-test. A statistically significant difference was found between the post-intervention and control groups in the scores of the economic behaviors sub-dimension (p<0.05) (Table 1).

**Table 1:** Distribution of pre-test and post-test scores of the Pro-Environmental Behavior Scale and its subdimensions in the intervention and control groups.

		Pre-test	Post-test	Within-group difference	
		Mean±SD	Mean±SD	t^b^	p
Environmental education subdimension					
	Intervention	13.84±3.07	15.50±3.03	-3.872	<0.001
	Control	14.19±2.57	12.73±2.70	4.733	<0.001
	Between-group difference	t^a^=-0.622 p=0.535	t^a^=4.905 p<0.001		
Economic behaviors subdimension					
	Intervention	10.13±2.56	11.36±2.27	-3.126	0.003
	Control	10.36±2.34	10.28±2.24	0.345	0.731
	Between-group difference	t^a^=-0.479 p=0.633	t^a^=2.430 p=0.017		
	Recycling subdimension				
	Intervention	7.53±3.27	10.65±2.96	-6.960	<0.001
	Control	8.50±3.03	8.67±2.86	-0.596	0.554
	Between-group difference	t^a^=-1.552 p=0.124	t^a^=3.468 p=0.000		
Pro-Environmental Behavior Scale					
	Intervention	31.51±7.06	37.51±6.68	-6.074	<0.001
	Control	33.05±5.90	31.69±5.67	2.661	0.010
	Between-group difference	t^a^=-1.205 p=0.231	t^a^=4.789 p<0.001		

t^a^: Between-group differences (independent samples t-test);

t^b^: Within-group differences (paired samples t-test). SD: standard deviation.

The mean scores of the Ecological Footprint Awareness Scale's food, transportation and housing, waste, and water consumption subscales decreased in the post-test compared to the pre-test, and there was no statistically significant difference between the intervention and control groups in the food subscale scores after the intervention (p>0.05). The mean scores of the energy subscale increased in the post-test compared to the pre-test, and no statistically significant difference was found between the intervention and control groups in the energy subscale scores after the intervention (p>0.05) (Table 2).

**Table 2:** Distribution of pre-test and post-test scores of the Ecological Footprint Awareness Scale and its subdimensions in the intervention and control groups.

		Pre-test	Post-test	Within-group difference	Within-group difference
		Mean±SD	Mean±SD	t^b^	p
Food subdimension					
	Intervention	23.84±5.03	21.67±10.70	1.414	0.163
	Control	22.17±3.68	21.67±3.63	1.588	0.119
	Between-droup difference	t^a^=1.933 p=0.056	t^a^=0.000 p=1.000		
Transportation and housing subdimension					
	Intervention	21.03±5.20	18.96±6.18	1.725	0.091
	Control	19.86±5.04	19.38±4.57	0.794	0.431
	Between-group difference	t^a^=1.167 p=0.246	t^a^=-0.397 p=0.692		
Energy subdimension					
	Intervention	32.28±9.43	32.53±11.63	-0.111	0.912
	Control	29.00±7.80	29.63±6.98	-1.321	0.192
	Between-group difference	t^a^=1.936 p=0.056	t^a^=1.543 p=0.127		
Waste subdimension					
	Intervention	21.40±7.31	19.50±7.13	1,322	0.192
	Control	19.65±5.32	19.51±4.86	0.406	0.687
	Between-group difference	t^a^=1.395 p=0.166	t^a^=-0.016 p=0.987		
Water consumption subdimension					
	Intervention	13.00±5.43	12.73±5.11	0.247	0.806
	Control	12.21±4.74	11.67±3.22	0.935	0.354
	Between-group difference	t^a^=0.788 p=0.432	t^a^=1.260 p=0.211		
Ecological Footprint Awareness Scale					
	Intervention	111.57±26.46	105.40±32.75	0.999	0.322
	Control	102.90±19.82	101.88±16.96	1.074	0.288
	Between-group difference	t^a^=1.891 p=0.062	t^a^=0.688 p=0.494		

t^a^: Between-group differences (independent samples t-test);

t^b^: Within-group differences (paired samples t-test). SD: standard deviation.

## DISCUSSION

The Environment-Based Education Program was associated with higher mean scores on the the Pro-Environmental Behavior Scale and its subdimensions (environmental education, economic behaviors, and recycling). This finding is consistent with previous studies reporting that environmental education programs enhance students’ pro-environmental behaviors^9,17,18^. Similarly, higher pro-environmental behavior tendencies observed among students in green-themed schools compared to public schools highlight the importance of the quality and implementation of education^19^. These results support the literature indicating that interventions aimed at improving environmental knowledge and attitudes contribute to better pro-environmental behaviors^20^. Additionally, prior environmental education has been shown to positively influence related subdimensions among high school students^10^. The consistency of these findings may be explained by developmentally appropriate educational approaches that support behavior change in adolescents. However, some studies report no significant effect on economic behaviors^21^, suggesting that environmental education should extend beyond knowledge transfer to include attitude and behavior-oriented practices. In this study, reinforcing learning through concrete examples, such as recycling practices, may have contributed to behavioral improvement.

In contrast, no significant change was observed in the Ecological Footprint Awareness Scale total and subdimension scores after the intervention. Accordingly, H0_2_, which stated that the Environment-Based Education Program would not create a significant difference in ecological footprint awareness between the intervention and control groups, could not be rejected. Although the intervention was effective in improving pro-environmental behaviors, it did not produce a measurable change in ecological footprint awareness. This finding contradicts studies indicating that environmental education improves ecological footprint awareness^13,22,23^. For instance, ecology-based education and 5E learning model applications have been shown to enhance awareness, particularly in food, energy, and water consumption^13,22^. The discrepancy may be related to the relatively short duration of the intervention, the characteristics of the study sample, and potential limitations in the sensitivity of the measurement tool to detect changes in adolescents’ ecological footprint awareness. In addition, some scale items may be more suitable for adults, which may have reduced the instrument's ability to capture changes in this age group.

Similarly, although increases in transportation, housing, energy, waste, and water consumption subdimensions have been reported in previous studies^12,13,22,23^, no significant changes were observed in the present study. This may be associated with adolescents’ limited autonomy in making decisions related to transportation, energy use, food consumption, and other environmentally relevant behaviors in daily life. Furthermore, the predominantly video-based educational approach may have provided insufficient opportunities for direct experience and behavior practice, which are considered important for developing ecological footprint awareness. Overall, the findings suggest that the intervention may have been too brief to produce measurable changes in ecological footprint awareness, or that the scale may have had limited sensitivity for detecting such changes among adolescents.

## CONCLUSION

In conclusion, it is understood that simply transferring information is insufficient in developing ecological footprint awareness; age, developmental characteristics, and opportunities for direct experience are decisive factors. Therefore, it is recommended that environmental education programs be structured in an age-appropriate, practice-based manner and linked to students’ daily life practices. Although the intervention group demonstrated a statistically significant increase in pro-environmental behavior scores, the magnitude of this change was limited, and participants remained within the "very low" score category. This finding suggests that statistically significant improvements may not necessarily reflect substantial behavioral change and highlights the need for longer-term and more intensive interventions.

### Limitations

This study has several limitations. First, the educational program was of short duration, and no long-term follow-up assessment was conducted; therefore, the sustainability of the observed effects over time could not be evaluated. Second, the sample size calculation was based on an effect size obtained from a study conducted with pre-service preschool teachers rather than adolescents, as no previous experimental study using the Ecological Footprint Awareness Scale in adolescent populations was available. Although the most comparable educational intervention was used for this purpose, this may have affected the precision of the sample size estimate. Third, the internal consistency of the economic behaviors subdimension was below the commonly accepted threshold; therefore, findings related to this subdimension should be interpreted with caution. Finally, multiple statistical comparisons were conducted without a formal adjustment for multiple testing, which may have increased the risk of Type I error. Future studies should include longer follow-up periods (e.g., 3- or 6-month assessments) to evaluate the persistence of changes in pro-environmental behaviors and ecological footprint awareness.

## Data Availability

The datasets generated and/or analyzed during the current study are available from the corresponding author upon reasonable request.
